# Mycobacterium tuberculosis resisters despite HIV exhibit activated T cells and macrophages in their pulmonary alveoli

**DOI:** 10.21203/rs.3.rs-3889020/v1

**Published:** 2024-01-31

**Authors:** Erwin Schurr, Monica Dallmann-Sauer, Vinicius Fava, Stephanus Malherbe, Candice McDonald, Marianna Orlova, Elouise Kroon, Aurélie Cobat, Stéphanie Boisson-Dupuis, Eileen Hoal, Laurent Abel, Marlo Möller, Jean-Laurent Casanova, Gerhard Walzl, Nelita du Plessis

## Abstract

To understand natural resistance to *Mycobacterium tuberculosis* ( *Mtb* ) infection, we studied people living with HIV (PLWH) in an area of high *Mtb* transmission. Given that alveolar leukocytes may contribute to this resistance, we performed single cell RNA-sequencing of bronchoalveolar lavage cells, unstimulated or *ex vivo* stimulated with *Mtb* . We obtained high quality cells for 7 participants who were TST & IGRA positive (called LTBI) and 6 who were persistently TST & IGRA negative (called resisters). Alveolar macrophages (AM) from resisters displayed more of an M1 phenotype relative to LTBI AM at baseline. Alveolar lymphocytosis (10%-60%) was exhibited by 5/6 resisters, resulting in higher numbers of CD4 ^+^ and CD8 ^+^ *IFNG* -expressing cells at baseline and upon *Mtb* challenge than LTBI samples. Mycobactericidal granulysin was expressed almost exclusively by a cluster of CD8 ^+^ T cells that co-expressed granzyme B, perforin and NK cell receptors. For resisters, these poly-cytotoxic T cells over-represented activating NK cell receptors and were present at 15-fold higher numbers in alveoli compared to LTBI. Altogether, our results showed that alveolar lymphocytosis, with increased numbers of alveolar *IFNG* -expressing cells and CD8 ^+^ poly-cytotoxic T cells, as well as activated AM were strongly associated with protection from persistent *Mtb* infection in PLWH.

### Introduction

In 2022, an estimated 7.5 million incident cases of tuberculosis (TB) were reported globally making it the highest number of newly diagnosed cases since 1995[1]. Globally, 6.3% of incident cases were people living with HIV (PLWH)[1]. Relative to HIV-negative persons, PLWH have a higher risk to develop clinical TB disease making TB in PLWH a major public health challenge in areas of high HIV prevalence[1–3]. In Southern Africa, more than 50% of people who fell ill with TB in 2022 were PLWH[1].

Exposure to *Mycobacterium tuberculosis* (*Mtb*), the cause of TB, leads to a spectrum of clinical manifestations ranging from absence of immunological or clinical features to life threatening TB disease[4–6]. Among exposed persons with no clinical symptoms, differences in innate immune response[7–9], *Mtb*-specific antibody production[10–14], interferon-γ (IFN-γ)-independent T cell responses[13–15] and *Mtb*-specific $CD4^+$ T cell immunity[16] indicate the complexity of *Mtb* infection control. The clinical and public health standards of established *Mtb* infection are provided by the tuberculin skin test (TST) and IFN-γ release assays (IGRA)[17]. The two tests measure different aspects of $CD4^+$ and $CD8^+$ T cell-dependent immunity in the periphery[17,18]. People who despite documented exposure remain persistently negative in both assays are “resisters” to IFN-γ conversion [19–22] while those with positive test results and no clinical evidence of TB disease are diagnosed with “latent TB infection (LTBI)” [23].

The identification of persons who are resistant to establishment of *Mtb* infection is complicated by the need to quantitate exposure and the lack of tools for direct, early-stage detection of *Mtb* in the lung. Moreover, the assays used to infer infection are conducted in peripheral blood and provide readouts of unknown relevance for protective immune responses in the lung. For example, considering the wellestablished role of IFN-γ in anti-mycobacterial immunity[24,25], it is counter-intuitive that a strong IFN-γ response to *Mtb* antigen is taken as evidence for failure of effective immunity. Despite these challenges, understanding the host resistance mechanisms that negate establishment of a pulmonary *Mtb* infection is of critical importance to derive interventions that prevent TB disease and transmission of *Mtb*.

To address this need in the context of HIV-TB, we focused our study on cells obtained from PLWH by bronchoalveolar lavage (BAL) from resister and LTBI participants from a region with high HIV-TB prevalence[12]. By performing single-cell RNA sequencing (scRNA-seq), we found striking differences in BAL cells at baseline and their response to *Mtb* challenge between resisters and LTBI. BAL samples from resisters were highly enriched for lymphocytes including subpopulations of $CD4^+$ and $CD8^+$ tissue resident memory (TRM) T cells, and a cluster of mycobactericidal poly-cytotoxic (*GNLY/GNZB/PRF1*$^{high}$) $CD8^+$ T cells expressing a suite of natural killer (NK) cell receptors. Resister alveolar lymphocytes presented higher counts of *IFNG* transcripts constitutively and after *ex vivo Mtb* challenge. Resister alveolar macrophages (AM) showed a pronounced shift towards a classically activated M1 phenotype. At 24h post *Mtb* challenge, transcripts for MICA and its activating NK receptor NKG2D (*KLRK1*) were strongly over-represented in AM and in poly-cytotoxic $CD8^+$ T cells of resisters, respectively. Combined, our data showed a strong association of mycobactericidal poly-cytotoxic $CD8^+$ T and activated AM cells with resistance to *Mtb* infection in PLWH as determined by IGRA and TST.

### Results

## Cell-type distribution of BAL cells

Our study was restricted to PLWH on long-term anti-retroviral therapy (ART) with no history of TB despite long-term exposure to *Mtb* (Fig. 1a). The 14 participants belonged to two well defined phenotypic groups of equal size: participants classified as “LTBI” who tested IGRA positive and displayed a TST ≥ 10 mm, and participants coined “resisters” who persistently tested IGRA negative with a TST = 0 mm (Fig. 1a and Table 1)[12]. All participants agreed to undergo a BAL and the recovered cells were kept unstimulated or challenged with *Mtb* for 6h and 24h. We performed scRNA-seq to investigate the BAL cellular composition, gene expression levels in the absence of *Mtb* and the transcriptomic responses to *Mtb* challenge (Fig. 1a). After quality control resulting in exclusion of one resister and data integration, we obtained single-cell transcriptome results for 257,671 BAL cells from six resister and seven LTBI participants (**Supplementary Table 1**). Based on gene expression we found two main subsets of cells (Fig. 1b). Alveolar macrophages (AM) and dendritic cells (DC) constituted the largest subset corresponding to 89% of the cells while the remaining 11% of BAL cells consisted of lymphocytes (T, B and NK cells) (Fig. 1b-c). However, BAL cells comprised strikingly different proportions of myeloid and lymphoid cells between the two groups, where resisters presented a significantly higher proportion of lymphocytes ($P = 0.0023$, Fig. 1d-e). While all LTBI subjects had < 5% of lymphocytes in their BAL samples (mean 2.93%), BAL samples from resisters presented a large spread of lymphocyte proportions ranging from 4–62.5% (mean 24.78%, Fig. 1e and **Extended Data** Fig. 1). None of the clinical or demographic variables collected, correlated with the degree of lymphocytosis. We noted minor peripheral blood contamination in the BAL of three samples from both groups, which had no correlation with lymphocytosis (**Supplementary Table 1**). We also obtained peripheral blood mononuclear cells (PBMCs) from the same participants and found no significant differences in lymphocyte proportions ($P = 0.61$, Fig. 1f) or cell subpopulations in PBMC between the two groups (**Supplementary Table 2**).

Table 1
Demographic and clinical data from the participants.

| Subject* | Group | Sex | Age (yrs) | ART time (yrs) | ART | IGRA | TST (mm) | HIV viral load (cp/mL) |
|---|---|---|---|---|---|---|---|---|
| 2RTB0014 | LTBI | M | 51 | 14 | TDF-FTC-EFV | Positive | 10 | 20 |
| 2RTB0092 | LTBI | F | 42 | 11 | TDF-FTC-EFV | Positive | 20 | 20 |
| 2RTB0113 | LTBI | F | 51 | 13 | TDF-FTC-EFV | Positive | 16 | NA |
| 2RTB0148 | LTBI | F | 47 | 16 | TDF-FTC-EFV | Positive | 20 | 0 |
| 2RTB0196 | LTBI | F | 59 | 11 | TDF-FTC-EFV | Positive | 17 | 242 |
| 2RTB0205 | LTBI | F | 53 | 12 | TDF-FTC-EFV | Positive | 22 | 0 |
| 2RTB0215 | LTBI | F | 43 | 17 | TDF-FTC-EFV | Positive | 18 | 0 |
| 2RTB0058 | Resister | F | 55 | 8 | TDF-FTC-EFV | Negative | 0 | 0 |
| 2RTB0062 | Resister | F | 47 | 16 | TDF-FTC-EFV | Negative | 0 | 58 |
| 2RTB0183 | Resister | F | 41 | 14 | TDF-3TC-ATV/r | Negative | 0 | 0 |
| 2RTB0209 | Resister | F | 40 | 10 | TDF-FTC-EFV | Negative | 0 | 20 |
| 2RTB0224¶ | Resister | F | 49 | 15 | TDF-FTC-EFV | Negative | 0 | 0 |
| 2RTB0253 | Resister | F | 54 | 12 | ABC-3TC-NVP | Negative | 0 | 22 |
| 2RTB0269 | Resister | F | 57 | 11 | TDF-FTC-EFV | Negative | 0 | 20 |

3TC: Lamivudine; ABC: Abacavir; ATV/r: Atazanavir/ritonavir; cp: copies; EFV: Efavirenz; F: female; FTC: Emtricitabine; LTBI: latent tuberculosis infection; M: male; NA: not available; NVP: Nevirapine; TB: tuberculosis; TDF: tenofovir; yrs: years.

* All participants are non-smokers. Participants are from Xhosa ethnic group, except 2RTB0113 and 2RTB0205 who are from Sotho.

¶ Sample excluded due to high proportion of dead cells in BAL scRNA-seq libraries (> 70%).

#### Characteristics of lymphocyte subpopulations in the absence of Mtb

To better define the differences in BAL cell subpopulations between resister and LTBI samples, we re-integrated and clustered the myeloid and lymphoid cells separately. Re-integration and clustering were done with all the infected and non-infected samples at the two time-points. Among lymphocytes we identified 19 clusters (Fig. 2a, **Extended Data** Fig. 2 and **Supplementary Table 3**). The majority of lymphocyte clusters comprised T cells ($CD3^+$), including $CD4^+$ naïve T cells (*CCR7*, *SELL* [CD62L]), $CD4^+$ regulatory T cells (*FOXP3*, *CTLA4*), $CD8^+$ cytotoxic T cells (*GZMs*), and $CD4^+$ and $CD8^+$ TRM expressing tissue-resident (TR) markers (*ITGA1* [CD49a], *ITGAE* [CD103], *CXCR6* and *CD69*) (Fig. 2a-c and **Extended Data** Fig. 2). We also detected one cluster of NK cells (*KLRC2*, *NCAM* [CD56]) and one B cell cluster (*MS4A1, CD79* and CD19) (Fig. 2a-c and **Extended Data** Fig. 2). For each participant, we determined the proportion of the lymphocyte subpopulations relative to their total lymphocyte count from the 6h non-infected samples. We compared these proportions between resister and LTBI participants using a Wilcoxon test and failed to detect significant differences (Fig. 2d). Similarly, we found no significant group differences in the ratio of $CD4^+$ to $CD8^+$ T cells in PBMC or BAL samples (median CD4/CD8 of 1.19 vs 1.13 in PBMC, and 0.51 vs 0.52 in BAL from resister vs LTBI, **Supplementary Table 2**). We also compared the proportions of lymphocyte clusters relative to the whole BAL which showed higher proportions of all resister clusters relative to LBTI BAL samples (**Extended Data** Fig. 3). Hence, differences in subpopulation proportions and CD4/CD8 ratios were not associated with lymphocytosis in the resister group.

We then compared the transcriptomic profile of BAL lymphocytes from resister to LTBI BAL samples in the absence of *ex-vivo Mtb* challenge. The low T cell counts in LTBI samples precluded the use of a comprehensive pseudobulk differential expression (DE) analysis of lymphocyte clusters. Hence, on the single cell level we compared the expression by the 6h non-infected lymphocytes from the two groups for the genes that encode IFN-γ and antimicrobial peptides which are key effectors of T cell anti-mycobacterial immunity [26–28]. Due to lymphocytosis, we found a significant larger numbers of *IFNG*-positive cells for the resister group across all clusters (Fig. 2e). Among resisters, the clusters with the largest proportion of *IFNG*-positive cells were L.3 (*GZMB*$^{high}$ $CD8^+$ T cell) and L.14 (*FOS*$^{high}$ $CD8^+$ T cell), with the latter cluster expressing *CD69* and various heat shock protein (*HSP*) genes. The cells in the L.14 cluster not only displayed higher expression levels of *IFNG* but the proportion of *IFNG*-positive cells in resisters was significantly larger relative to LTBI (Fig. 2e). Finally, we determined the transcript counts of antimicrobial peptides granulysin (*GNLY*), granzyme B (*GNZB*) and perforin (*PRF1*)[27,28]. We detected one cluster, L.8, co-expressing the three genes at baseline (Fig. 2f). Since the cells were CD3 and CD8 positive, we annotated the L.8 cluster as $CD8^+$ poly-cytotoxic T cells (Fig. 2b, **Extended Data** Fig. 2 and **Supplementary Table 3**). In L.8, *GZMB* and *PRF1* were expressed at approximately the same level in cells from the resister and LTBI participants, while *GNLY* was detected with higher expression in the resister cells (Fig. 2f). Across all clusters, resister lymphocytes constitutively expressed higher counts of *IFNG*, *GZMB* and *GNLY* transcripts relative to LTBI.

### Characteristics of alveolar macrophages in the absence of ex vivo Mtb challenge

Next, we annotated the subpopulations in the AM/DC subset where we identified 12 clusters (Fig. 3a and **Supplementary Table 3**). Of these, one small cluster (DC.9) consisted of DC, while all remaining clusters were subpopulations of macrophages (Fig. 3a-c). All macrophages expressed markers that were consistent with tissue-resident AM (*MARCO*, *PPARG*, *FABP4*) except for cluster MoAM.4 which we annotated as infiltrating monocyte-derived macrophages (*CCL2*, *CSFR1*, *MMP9* and CD14) (Fig. 3b and **Extended Data** Fig. 2). We found no significant differences in the proportions of tissue-resident AM or infiltrating monocyte-derived macrophages between cells from resister and LTBI participants (Fig. 3d).

To analyze the transcriptomic profiles of these myeloid BAL cell populations, we performed pseudobulk DE analysis between cells from resister and LTBI participants in the absence of *Mtb* challenge. The DE analyses were done for each cluster independently, excluding AM.10, and AM.11 due to their low number of cells per library. For the nine AM clusters and the single DC cluster, we detected a total of 4,275 genes (comprised of 2,167 distinct genes) that were differentially expressed between resister and LTBI cells (Fig. 4a-b, **Extended Data** Fig. 3 and **Supplementary Table 4**). Strikingly, only the differentially expressed genes (DEG) with higher expression in resister cells resulted in enrichment of GO-terms/pathways (Fig. 4c and **Supplementary Table 5**). For example, AM from the resister group presented higher expression of genes for pathways related to oxidative phosphorylation as well as cytokine-, chemokine- and interleukin-mediated signaling, with the most pronounced differential gene expression in AM.3 (*ERRFI1*$^{high}$ TR-AM) and MoAM.4 cells (Fig. 4c).

Next, we investigated the extent to which differential baseline gene expression reflected changes in transcription factor (TF) activities. TF activity was inferred based on the gene expression of target genes induced or repressed by the TFs. For the TF regulatory network analysis, we calculated TF activity scores using the genes differently expressed between resisters and LTBI samples in the absence of *Mtb* (Fig. 4d and **Supplementary Table 6**). In AM, we found significant differences in TF activities between the groups for TFs involved in M1 and M2 macrophage polarization. For example, TFs AP1, NFKB, CEBPG and IRF1 that are linked to an M1-state showed stronger activity in AM from resisters (Fig. 4d). Similarly, we found higher expression of M1 genes such as *IL6*, *CCL3* and *IL1B* as well as the lower expression of the canonical M2 marker *CD163* in AM from resister compared to LTBI samples (Fig. 4e). This showed that alveolar macrophages from resisters were shifted towards an M1 transcriptomic profile in the absence of *Mtb.*

When we repeated the baseline comparison of resister vs LTBI in AM removing the effect of lymphocyte proportion from the model, we observed that this adjustment differently affected AM/DC clusters (**Extended Data** Fig. 4). More strikingly, while we still observed DEG between resister and LTBI cells, the number of DEG was small and the genes were enriched only in few GO-terms/pathways (**Extended Data**

Fig. 4). This suggested that the vast majority of the AM/DC functional transcriptomic differences observed between resisters and LTBI were correlated with alveolar lymphocytosis.

#### Cell-cell communication in the absence of ex vivo Mtb challenge

We then investigated if the resister and LTBI phenotypes were reflected in an altered crosstalk between cell populations during short-term *in-vitro* culture. For that, we performed a cell-cell communication analysis of the non-infected cells with 6h of incubation by mapping the expression of receptor-ligand pairs across the BAL cell clusters from the resister and LTBI samples. We found that cell subpopulations from the resister group displayed more and stronger cell-cell interactions (**Extended Data** Fig. 6a). In both the resister and LTBI groups, AM presented a higher number of cell-cell interactions as senders (expressing the ligands) and receivers (expressing the receptors) than DC and lymphocytes (Fig. 5a). However, all AM clusters in the resister samples presented higher numbers of cell-cell communications than in the LTBI, a trend which was observed to a lesser extent in the lymphocyte clusters (Fig. 5a). When we further evaluated the cellular crosstalk, we observed a set of signaling pathways defined by different cell-cell interaction between resister and LTBI samples (**Extended Data** Fig. 6b). Consistent with the significantly higher number of *IFNG*-expressing cells in the absence of the *ex vivo Mtb* challenge (Fig. 4f-g), cell-cell communication for the IFN-γ signaling pathway was exclusively detected in the cells from the resisters (**Extended Data** Fig. 6b). In resisters, L.3 and L.14 presented significant cell-cell interactions as senders (expressing *IFNG*) with the myeloid cells as the receiver clusters (expressing *IFNGR1* + *IFNGR2*) (Fig. 5b). TNF was mostly expressed in myeloid cells, but also in cluster L.3 (Fig. 5c-d). TNF receptor 1 (*TNFRSF1A*) was highly expressed only in AM, while TNF receptor 2 (*TNFRSF1B*) was found in both myeloid and lymphoid clusters (Fig. 5d). However, expression of *TNFRSF1B* was most pronounced in the MoAM.4 and DC.9 clusters which are not classical tissue-resident AM. There was a non-significant trend of higher expression of *TNFRSF1A* among resister macrophages (Fig. 5d). This might explain the higher number of cell-cell interactions within the TNF crosstalk in resister vs LTBI cells, especially the communications mediated by *TNFRSF1A* (**Extended Data** Fig. 6c). The crosstalk between TNF and *TNFRSF1B* was dominated by the higher T cell counts in resister lymphocyte clusters (Fig. 5d and **Extended Data** Fig. 6d). In summary, there was higher cell-cell crosstalk in resisters for INF-γ and TNF signalling relative to LTBI.

#### Alveolar macrophage response to ex vivo Mtb challenge

We next investigated the transcriptomic response of BAL cells after 6h and 24h of *ex-vivo* challenge with *Mtb.* Given the size of our study sample, we focused the analysis on established mycobactericidal mechanisms of human cells (**Supplementary Table 7**). In the myeloid cells, these were the antimicrobial peptide cathelicidin (*CAMP*), the defensins as well as TNF, which can mediate the killing of *Mtb* via induction of reactive oxygen species (ROS)[29–31]. Only one defensin gene, Defensin beta 1 (*DEFB1*), was expressed in the BAL cells. *DEFB1* and *CAMP* were transcribed only by the tissue-resident AM and displayed reduced transcription with time in culture (**Extended Data Fig. 7a**). *CAMP* presented no significant change in expression after *Mtb* infection. In contrast, resister cells of clusters AM.3, AM.7

(Activated TR-AM) and AM.8 (*ANXA1*$^{high}$ TR-AM) exhibited a small but significant higher expression of *DEFB1* over LTBI cluster cells at 24h of *Mtb* infection (**Extended Data Fig. 7a**). For *TNF* transcription, we observed significantly increased transcription at 6h post-infection (p.i.) for resister macrophages over LTBI cells in clusters AM.0 (*ASH1L*$^{low}$ TR-AM), AM.2 (*PEX14*$^{high}$ TR-AM), AM.3, MoAM.4 and AM.11 (proliferating AM) (Fig. 6a). TNF transcription dropped substantially across all clusters at 24h but remained significantly higher in resister-derived cells for cluster AM.3 (Fig. 6a). Hence, while the TNF transcriptional response was consistently stronger for resister AM this superior TNF response was more pronounced at the early phase of *Mtb* infection.

### Alveolar lymphocyte response to ex vivo Mtb challenge

When assessing the transcriptomic *IFNG* response of alveolar lymphocytes, we noticed a significant response to *Mtb* in cluster L.3 (*GZMB*$^{high}$ CD8$^{+}$ cytotoxic T) by LTBI cells with stronger response observed at 6h (Fig. 6b). We did not observe a similar *IFNG* induction in any of the resister clusters. However, the baseline count of *IFNG* transcripts in L.3 resister cells was higher than the stimulated *IFNG* count in LTBI samples at both 6h and 24 h p.i. (Fig. 6b). Across the remaining T cell clusters, at 6h p.i. we observed significantly higher numbers of cells expressing *IFNG* transcripts in resister vs LTBI samples (Fig. 6b). Notable were L.14 (*FOS*$^{high}$ CD8 + T) cells where resisters expressed higher levels of *IFNG* transcripts and a significantly larger proportion of cells were *IFNG*-positive compared to LTBI samples (Fig. 6b).

A main interest for our analyses were the expression changes in response to *Mtb* challenge of the mycobactericidal peptides *GNLY*, *GNZB* and *PRF1*. Irrespective of *Mtb* challenge, only the CD8$^{+}$ poly-cytotoxic T cells from cluster L.8 (Poly-cytotoxic CD8 + T [*GZMB*/ *GNLY*/ *PRF1*$^{high}$]) co-expressed all three genes (Fig. 2f and Fig. 6c). In L.8 cells from resister and LTBI samples, *GNLY* was induced to similar levels in both groups by *Mtb* infection (Fig. 6c). Similarly, *GZMB* was expressed at approximately the same level at 6h and 24h p.i. in resister and LTBI L.8 cells (Fig. 6c). Perforin showed a trend for higher expression in LTBI samples at 6h after *Mtb* challenge. However, at 24h *PRF1* was expressed at the same level in a larger proportion of resister cells (Fig. 6c). Moreover, we noticed that the poly-cytotoxic CD8$^{+}$ T cells from L.8 also expressed the genes for the NK activating receptors NKG2D (*KLRK1*) and NKG2C (*KLRC2*) as well as for the inhibitory receptor NKG2A (*KLRC1*) and for CD94 (*KLRD1*) required for the CD94/NKG2 complex (Fig. 2c and **Supplementary Table 3**).

*KLRD1* was expressed at approximately the same level at 6h and 24h p.i. in both groups. Similarly, the *KLRC1* gene encoding the inhibitory NKG2A receptor was expressed at approximately the same low levels at 6h and 24h after *Mtb* infection in both groups (Fig. 6c). Conversely, the genes encoding the activating receptors, *KLRC2* and *KLRK1*, were expressed at higher levels in a larger proportion of L.8 cells by resisters. This was most pronounced for *KLRK1* where at 24h p.i > 60% of L.8 cells in resisters expressed the gene vs only 20% in LTBI cells (3-fold difference, Fig. 6c). Overall, the ratios of activating and inhibitory receptors demonstrated a strong switch in favour of activation of the CD8$^{+}$ poly-cytotoxic T cells in resisters. Even more striking, the numbers of L.8 cells in BAL samples were significantly different between resisters and LTBI samples ($P = 0.0009$). The mean ratio of the $CD8^+$ poly-cytotoxic T cells was 0.077% of all BAL cells for the LTBI group and 1.2% for the resister group, presenting an over 15-fold increase in this group over LTBI (Fig. 6c).

The heterodimers NKG2A-CD94 (*KLRC1* + *KLRD1*) and NKG2C-CD94 (*KLRC2* + *KLRD1*) interact with HLA-E, while NKG2D (*KLRK1*) interacts with the non-classical MHC class I ligands MICA and MICB[32,33]. In our data, *HLA-E* was highly expressed in all AM/DC clusters and *HLA-E* expression was significantly induced by 24h of *Mtb* challenge to a similar extent in both groups (**Extended Data** Fig. 6b). *MICA* and *MICB* genes were transcribed by macrophages with higher expression at the 24h p.i. time-point (**Extended Data** Fig. 6c). *MICB* presented lower expression than *MICA* with similar levels by both groups. Conversely, at 24h *MICA* expression was increased in seven AM clusters from resisters over LTBI participants (Fig. 6d). Except cluster AM.10, the remaining six clusters expressed *MICA* in a higher proportion of resister cells (mean 34% vs 26.5%) at significantly higher levels (**Supplementary Table 7**). However, differences in expression levels were overall modest with $\log_2 FC < 0.1$ (**Supplementary Table 7**). The most pronounced difference was found in AM.3 where a 1.5-fold higher proportion of infected cells expressed *MICA* transcripts in resister vs LTBI cells with $\log_2 FC = 0.125$ (Fig. 6d, **Supplementary Table 7**). Combined, this supported the NKG2D (*KLRK1*) – MICA receptor ligand interaction as critical feature for recognition of infected AM by poly-cytotoxic $CD8^+$ T cell.

## Discussion

We performed a single cell transcriptomic study of BAL cells obtained from persons who had previously been identified in an extensive study of the "resister" phenotype[12]. By combining BAL sampling with scRNA-seq methodology in this unique population, we uncovered the novel finding that resister PLWH in a high TB risk area display airway lymphocytosis with heightened baseline expression of *IFNG* by both $CD4^+$ and $CD8^+$ T cells, despite long-term IFN-γ unresponsiveness to *Mtb* antigens in peripheral blood. The key role of IFN-γ in anti-mycobacterial immunity has been unambiguously established[24,26,34]. This led to the paradoxical situation where persons were classified as resisting *Mtb* infection, and hence tuberculosis, on the basis that they did not mount an *Mtb*-triggered IFN-γ response by peripheral blood mononuclear cells. Here we show that resister alveolar T cells constitutively produce *IFNG* transcript levels which exceed those in LTBI T cells even following *Mtb* stimulation. It is reasonable to assume that this excess of *IFNG* transcripts will manifest as higher secretion of IFN-γ by resister T cells compared to LTBI cells in the lung at the very early phase of infection before infiltration of immune cells occurs. Similarly, it is likely that the observed shift of resister AM towards an M1 macrophage phenotype is related to the high constitutive presence of IFN-γ in resister alveoli.

The basic premise of our study was that BAL cells from resister and LTBI persons differ in their anti-*Mtb* capacity. We therefore focused the transcriptional analysis of BAL cells on host responses that have been shown to kill *Mtb*. Two potent signaling molecules that directly increase the anti-microbicidal activity of human macrophages are IFN-γ[35] and TNF[36]. Transcription of both effectors constitutively and after *Mtb*

challenge was significantly higher in resister BAL samples. IFN-γ and TNF have been associated with several potential anti-*Mtb* cellular responses such as increased lysosome acidification, increased autophagy or heightened ROS production[37,38]. It is not certain how the lysosomal environment mediates killing of *Mtb* given the resistance of *Mtb* to low pH and a possible cytoplasmic escape of the bacilli[39,40]. Similarly, it is not certain that autophagosomes can facilitate killing of *Mtb* in the absence of IL-26 which was not expressed by BAL cells[41]. In contrast, ROS have been shown to directly kill *Mtb* and the respiratory oxidative burst by human macrophages is a key mechanism by which invading pathogens, including *Mtb*, are killed[42,43]. The key role of ROS in killing of *Mtb* is shown by the increased susceptibility of chronic granulomatous disease (CGD) patients to TB[44–46]. CGD patients carry loss of function mutations in any of the five subunits of NADPH oxidase resulting in the inability to mount a respiratory burst against infectious pathogens. Similarly, patients with mutations in the CYBB subunit of NADPH oxidase show exceptional susceptibility to TB[47]. We therefore concluded that the likely main effect of IFN-γ and TNF on resister AM was a rapid and more efficient ROS response to *Mtb* as compared to AM from LTBI samples.

By investigating a possible contribution of antimicrobial peptides in the killing of *Mtb*, we identified a $CD8^+$ T cell cluster that co-expressed granulysin (*GNLY*), granzyme B (*GZMB*) and perforin (*PRF1*). Granulysin had previously been shown to kill *Mtb* by altering membrane permeability of the bacillus and granulysin levels correlated with treatment success of TB[28,48–51]. In leprosy, presence of granulysin in skin lesions correlated with protection from disseminated forms of the disease[52]. In our data, the distribution of *GNLY* expressing cells was focused on lymphocyte clusters L.8 and L.18 of which the latter is a small cluster annotated as classical NK cells. L.18 cells did not express *GZMB* which was, however, expressed by three other $CD8^+$ clusters. Granzyme B potentiates the anti-*Mtb* activity of granulysin. It was shown for multiple bacterial species that granulysin delivers the protease granzyme B to the bacteria resulting in rapid bacterial death[27,52]. In addition to the direct microbicidal effect on *Mtb*, granzyme B triggers apoptosis of pathogen-infected host cells and cleaves bacterial enzymes such as superoxide dismutase and catalase which protect *Mtb* from ROS activity. Hence, the proteolytic activity of granzyme B might synergize with the ROS response expected to be stronger in resister macrophages to damage the bacilli that might otherwise withstand ROS action. Finally, expression of *PRF1* was limited to cluster L.8. Perforin punctures holes in host cell membranes and boosts the killing of intracellular pathogens such as *Mtb* by facilitating the entry of granzyme B and granulysin into host cells[53].

Poly-cytotoxic $CD8^+$ T cells had previously been shown to inhibit the growth of *Mtb*[54] and these cells were able to effectively kill three intracellular parasites[55]. Infliximab-triggered elimination of granulysin and perforin expressing CD8 + T cells from the blood circulation was associated with increased TB incidence in patients with rheumatoid arthritis[56]. Moreover, poly-cytotoxic $CD8^+$ T cells expressing the activating NKG2C (*KLRC2)* NK cell receptor are enriched in the tuberculoid form of leprosy and can act independently of TCR to trigger release of their anti-microbial cytotoxic granules by interacting with HLA-E molecules on target cells[57]. Results from the macaque model of tuberculosis strongly support the role of poly-cytotoxic $CD8^+$ T cells in TB resistance. Specifically, $CD8^+$ T cells expressing granulysin, granzymes and perforin were associated with protective granuloma in macaques[58] and linked with protection in the early stage of *Mtb* infection[59]. In resisters, $CD8^+$ poly-cytotoxic T cells (cluster L.8) were present on average at 15-times larger numbers in alveoli, with an estimated 3-fold higher proportion of these cells expressing *KLRK1* which encodes the activating NKG2D receptor that recognizes MICA as ligand on target cell. Combined with the observation that *MICA* transcripts were expressed at higher levels in a larger number of AM cells in resister compared to the LTBI samples, this provided a strong case that cytotoxic mechanisms are a main effector of increased resistance to infection with *Mtb*. These findings strengthen the long-held view that absence of peripheral T cell immunity detected by TST and IGRA is a good correlate for absence of established infection with *Mtb*. Our data strongly support the notion that resisters have an increased capacity to kill alveolar *Mtb.* Nevertheless, it is likely there will be a period of transient infection where *Mtb* and *Mtb*-derived antigens are present in alveoli. If and how this could give rise to B cell and IFN-γ independent responses while avoiding classical $CD4^+$ based T cell immunity is not known.

While not directly linked to killing of *Mtb*, we did identify heterogenous subsets resembling $CD4^+$ (L.0) and $CD8^+$ (L.4) TRM. It remains to be investigated if the L.0 cluster is derived from $CD4^+$ mucosal associated invariant T (MAIT) cells. TRM cells are located at pathogen entry portals and persist locally at mucosal tissue sites where they provide defense against pathogens such as *Mtb*[60,61]. TRM cells are poised to deliver a faster and more robust response upon re-exposure to a pathogen and promote the generation of antibodies[62]. In fact, a subpopulation of $CD4^+$ TRM cells which are colocalized with B cells in Inducible Bronchus-Associated Lymphoid Tissue (iBALT), promote local antibody production and enhance $CD8^+$ TRM cells via IL-21 production[63,64]. Further studies are required to analyze the functional properties of the various T cell subsets described here, including comprehensive flow cytometric analysis of airway TRM cells.

Our study was conducted in a sample of PLWH. It is possible that in HIV-negative people different mechanisms are at play that interfere with immune conversion or mediate *Mtb* infection resistance. In a recent study, baseline gene expression and the transcriptomic response of monocytes to *Mtb* differed between $HIV^+$ and $HIV^-$ donors[9]. However, the inflammatory monocytes-derived cells (MoAM.4) detected among the BAL cells in our study did not show transcriptional evidence for disturbances in lipid metabolism as observed in monocytes of HIV-negative resisters. Similarly, we failed to detect expression of subunits of the AMPK regulator of cellular metabolism, previously shown to be associated with the resistance phenotype in peripheral blood in HIV-negative persons, in any of the BAL cell clusters[7]. These data suggested that mechanisms of host response to *Mtb* between PLWH and HIV-negative people might differ. On the other hand, the evidence implicating poly-cytotoxic $CD8^+$ T cells in protection from mycobacterial diseases had been obtained in HIV-negative persons and the results obtained in the macaque model argue against an HIV-driven bias in the detected effector cells[54,57]. While we identified a major effector mechanism of resistance, considering the large number of macrophage genes differentially expressed between the study groups, it is possible that additional host response mechanisms contribute to the resistance phenotype.

The proposed protection from *Mtb* is tightly correlated with the increased proportion of T cells in BAL samples from resisters. Moreover, differential gene expression between resister and LTBI AM was strongly dependent on percent of lymphocytosis. Hence, a central question is to what extent alveolar lymphocytosis is also found in HIV-negative resisters. It is unknown what genetic or environmental factors impact lymphoid or myeloid or both cell types to give rise to alveolar lymphocytosis. In PLWH, a possible environmental factor is HIV infection. Lymphocytic alveolitis is a common occurrence during early and mid-stage HIV infection[65]. The percentage of lymphocytosis is correlated with HIV pulmonary viral load and enriched for HIV specific $CD8^+$ cells[66,67]. However, such HIV-associated lymphocytic alveolitis improves with anti-retroviral treatment and viral control[68] and the participants in our study had been on long term ART with documented viral suppression. Similarly, the near identity of T cell populations in LTBI (no lymphocytosis) and resister T cells (lymphocytosis) argues against HIV-dependent lymphocytosis in the group of resisters. A recent scan of BAL cells from HIV-negative resisters and LTBI persons from Uganda did not detect evidence for lymphocytosis in the resister group[69]. Considering that lymphocytic alveolitis can be observed in 20–30% of healthy persons for unknown reasons[67,70] and that close house hold contacts had the highest BAL lymphocyte counts in a large comparative survey[71], a possible explanation could be that the infectious pressure experienced by Ugandan resisters was lower and selection for resisters with stronger lymphocytosis expression did not occur. Clearly, more detailed studies of BAL cells in different *Mtb* exposure settings are required to fully understand the range of the protective effects of poly-cytotoxic $CD8^+$ T cells for *Mtb* infection resistance. However, most excitingly, our results add to the growing evidence from animal and human studies that point to a critical protective role of poly-cytotoxic $CD8^+$ T cells and alveolar but not peripheral blood *IFNG* expression over the entire spectrum of TB pathogenesis and identify these cells as prime targets for future vaccine studies.

## Methods

## Study participants

The participants of this study are part of the ResisTB cohort, described in detail by Kroon *et al*[12] and Gutierrez *et al*[21]. All participants enrolled in the ResisTB study are PLWH with no history of TB while living in Cape Town, South Africa, an area of high *Mtb* transmission. The “resister” group, previously coined “HITTIN” (HIV-1-infected persistently TB, tuberculin and IGRA negative), is composed of subjects with three consecutive IGRA negative assays and a TST = 0 mm. The “LTBI” group, previously coined “HIT” (HIV-1-infected IGRA positive tuberculin positive), is composed of subjects with IGRA positivity in two consecutive tests and TST ≥ 10 mm (Table 1). All participants have a history of low peripheral $CD4^+$ T cell count (< 200/mm$^3$), which was reconstituted after anti-retroviral therapy (> 500/mm$^3$). For the present study, 14 participants (7 resisters and 7 LTBI) underwent a BAL procedure. Except for one LTBI participant, all participants were female. Participants were from the Xhosa ethnic group, except two LTBI individuals that were from the Sotho ethnic group. At the time of BAL collection, the mean (± standard deviation) age was 49 ± 6 years in the resister and 49 ± 5 years in the LTBI group (Table 1). All subjects were non-smokers. Bronchoscopies with BAL were performed according to current recommendations[72,73] in a research bronchoscopy facility (SU-IRG Biomedical Research Unit, Stellenbosch University) as recently described[71]. In brief, all participants were pre-screened for fitness for bronchoscopy according to predefined criteria by a study clinician with knowledge of the procedure. Active TB or other lung infections were excluded by chest X-ray, no lung parenchymal abnormalities were observed, and all tested negative by sputum GeneXpert Ultra and liquid culture. The bronchoscope was targeted to lung regions affording ease of accessibility and the lavage was performed by instilling sterile saline solution at 37°C up to a maximum volume of 240ml in aliquots of 60ml at a time, with aspiration between aliquots. Aspirated fluid was collected in sterile 50ml polypropylene tubes and transported on ice to the laboratory.

Research was performed in accordance with the Declaration of Helsinki and all participants provided written informed consent for the study procedures, which was approved by the Stellenbosch University (SU) Health Research Ethics Committee (N16/03/033), the SU Research Ethics for Biological and Environmental Safety Committee (BES-2023-19406) and the Research Institute of the McGill University Health Centre (MP-CUSM-15-406).

## Blood count

Blood was collected by phlebotomy in a heparinized vacutainers and PBMC isolated according to the standard Ficoll isolation method. PBMC were cryopreserved in 10% dimethyl sulfoxide (MilliporeSigma, Massachusetts, USA) and 90% fetal bovine serum (Cytiva, Massachusetts, USA). Differential counts were performed on PBMC by standard flow cytometry staining for the markers (CD45, CD3, CD4, CD8, CD19 and CD14).

## BAL cells collection

BAL fluid was placed on ice immediately after aspiration. Processing was initiated within two hours of collection. If the pellet was judged contaminated with blood by visual inspection, an additional red cell lysis step was performed in 1 ml of Lonza ACK lysis buffer (1x) [Whitehead Scientific (Pty) Ltd, SA]. The total cell count was conducted using a haemocytometer and viability check by Trypan Blue exclusion method. A fraction was used for a differential count by cytospin (Simport, Saint-Mathieu-de-Beloeil, Canada). BAL cells were cryopreserved in 10% dimethyl sulfoxide (MilliporeSigma, Massachusetts, USA) and 90% fetal bovine serum (Cytiva, Massachusetts, USA) and, by gradual cooling to − 80°C in a Nalgene Mr Frosty™ container [Sigma Aldrich (Pty) Ltd, Gauteng, South Africa] with isopropanol for 24 h followed by long term preservation in liquid nitrogen.

## BAL cell culture

Cryopreserved BAL cells were set in culture following a dropwise thawing protocol as follows. The content of a cryovial was thawed at 37°C in water bath for 2 minutes. Cell suspension was transferred into a sterile 50ml tube. Dropwise, 1ml of RPMI-1640 with L-glutamine (Gibco, USA) with 50% heat-inactivated fetal bovine serum (hiFBS) (Gibco, USA), 1% penicillin/streptomycin (Gibco, USA), 10mM HEPES (Gibco, USA), 1% non-essential amino acids (Gibco, USA) and 5µg/ml Amphotericin B (Gibco, USA), 20µg/ml DNAse I (Roche, Germany) was added to the cells reaching a total volume of 32ml. The tube was spun at 320g for 12 minutes at room temperature. Cell pellet was dislodged and resuspended for the second wash in 20ml of RPMI-1640 containing 20% hiFBS, 10mM HEPES (Gibco, USA), 1% non-essential amino acids (Gibco, USA) and 2.5µg/ml Amphotericin B (Gibco, USA), 20µg/ml DNAse I (Roche, Germany). Cell were seeded at $2.5\times10^5$ cells per well in 24 well-plates Nunclon Sphera Low-attachment (ThermoFisher, USA) in 500µl of culture medium (RPMI-1640 with L-glutamine (Gibco, USA), containing 10% human serum (heat-inactivated AB + off the clot, Wisent, Canada), 10mM HEPES (Gibco, USA), 1% non-essential amino acids (Gibco, USA) and 2.5ng/ml Amphotericin B (Wisent, Canada) and incubated at 37°C, 5% $CO_2$, and 95% relative humidity.

#### Mycobacterial cultures and BAL cell Mtb infection

Virulent *Mtb* strain H37Rv was grown in a liquid culture of Middlebrook 7H9 medium (BD Difco, USA) containing 0.2% glycerol (Fisher, USA), 0.05% Tween-80 (Sigma-Aldrich, USA) and 10% albumin-dextrose-catalase (BD, USA) at 37°C in rolling incubators. Bacteria were grown to log phase determined by an optical density of 0.6 to 0.8 at 600nm, prior to inoculum preparation. Further, bacterial cultures were spun for 15 minutes at 3700 rpm, resuspended in RPMI-1640 and dislodged with a 22G needle. Cell suspensions were filtered through 5µm filters (Millipore, USA) to ensure single mycobacteria suspensions for BAL cells challenge. Bacterial counts of inocula were done using disposable Neubauer hemocytometer (C-Chip, INCYTO, South Korea). Bacterial loads were confirmed by colony-forming unit (CFU) counts by plating serial dilution of inoculum in 7H9 growth medium on Middlebrook 7H10 agar (BD, USA) plates containing 0.5% glycerol and 10% oleic acid-albumin-dextrose-catalase (BD, USA). Colonies were counted 4 weeks post-plating. BAL cells were infected on average at a multiplicity of infection (MOI) of 6.5:1 for 6h and 24h at 37°C, 5% $CO_2$, and 95% relative humidity. In parallel, non-infected samples were incubated for the same periods.

## Single cell RNA library preparation and sequencing

After incubation, BAL cells were collected and washed once in cold PBS (Wisent, Canada) containing 1% bovine serum albumin (Wisent, Canada). Cell clumps were removed by passing cell suspension through 40µm FlowMi strainer (Bel-Art, USA). Single cell capture and library preparation was performed with Chromium Next GEM Single Cell 3   Reagents Kit v3.1 (10X Genomics, USA). Cell suspensions were loaded on a Chromium Next GEM Chip G (10X Genomics, USA) together with gel beads from Chromium Next GEM Single Cell 3   GEM Kit v3.1 and captured on Chromium Controller (10X Genomics, USA) with recovery target of $1\times10^4$ cells. cDNAs were generated following the 10X Genomics protocol CG000315 and their quality was checked with Bioanalyzer High Sensitivity DNA Kit (Agilent, USA). One quarter of the total cDNA was used to generate sequencing libraries using Library Construction Kit (10X Genomics, USA) and barcoded using Dual Index plate TT set A (10X Genomics, USA). Obtained libraries were double side size-selected using SPRIselect beads (Beckman Coulter, USA) to enrich for fragments 300–800 base pairs long, centered at 450bp. Libraries were checked for quality with Bioanalyzer High Sensitivity DNA Kit and paired-end sequenced on Illumina NovaSeq 6000 S4 flowcells aiming to obtain 50,000 reads per cell. We aimed for generating 56 scRNA-seq libraries from the 14 participants, which included libraries from four conditions per subject based on the infection status and time of incubation of the cells: i) *Mtb*-infected 6h, ii) non-infected 6h, iii) *Mtb*-infected 24h and iv) non-infected 24h (**Supplementary Table 1**). We successfully generated 55 scRNA-seq libraries, while one non-infected 6h library from an LTBI subject failed in the library preparation and was not sequenced (**Supplementary Table 1**).

## Surface markers staining for single-cell data analysis

To facilitate the characterization of BAL cell sub-populations of leukocytes, we performed CITE-seq (cellular indexing of transcriptomes and epitopes by sequencing) with non-infected cells from two participants (one LTBI and one resister), using a TotalSeq-B Human TBNK cocktail of monocyte-, T-, B-, NK, NKT-cell specific markers (BioLegend, USA). Following the 10X Genomics protocol CG000149_RevD, 1 μg of Antibody cocktail was used per $1\times10^6$ BAL cells in 100 μL staining volume. Using Chromium Controller, Chromium Next GEM chip G and Chromium Next GEM Single Cell 3 Reagents Kit v3.1 (10X Genomics, USA) cell emulsions, with a target capture of $1\times10^4$ cells, were obtained for scRNA-seq and Cell Surface Protein library preparations. Following the 10X Genomics protocol CG000317_RevD, cDNA and DNA from cell surface protein Feature Barcode were amplified using Feature cDNA Primers 2 from 3’ Feature Barcode kit (10X Genomics, USA). After amplification step, samples were size-selected with SPRIselect beads (Beckman Coulter, USA), where cDNA bound to beads, while DNA from Cell Surface Protein Feature Barcode remained in the solution. After magnetic separation, cDNA was eluted from beads and used for scRNA-seq libraries as described above, whereas supernatants were used for Cell Surface Protein Library construction. The Cell Surface Protein Feature Barcode DNA was purified by an additional round of SPRIselect beads precipitation and amplified with primers from the Dual Index plate NT set A (10X Genomics, USA). Library quality was assessed with Bioanalyzer High Sensitivity DNA Kit and paired-end sequenced on Illumina NovaSeq 6000 S4 flowcells aiming to obtain 10,000 reads per cell.

## Preprocessing and data integration

Combining the 55 scRNA-seq libraries (from the 6h and 24h *Mtb*-infected and non-infected samples) with the two CITE-seq samples (scRNA-seq plus cell-surface antibody capture), we generated 57 scRNA-seq libraries from the 14 subjects (**Supplementary Table 1**). Cell Ranger software v7.0.1 (10X Genomics, USA) was used for alignment to GRCh38 human genome and generation of feature-barcode matrices per library. Data analysis was done using Seurat v4.3.0 [74]. Seurat objects for each library were created using CreateSeuratObject function with min.feature = 300, and gene expressions were normalized using "LogNormalize" method from Seurat NormalizeData function with default setting. An initial annotation of main cell-types in the raw data was done for quality control and filtering. Annotation was based on gene expression of canonical markers for tissue-resident AM (*CD68, MARCO*), infiltrating monocyte-derived AM (*CD68, CSF1R, CCL2*), DC (*LAMP3, CCR7*), T/NK cells (*CD3D, TRAC, NKG7*), B cells (*CD79A, MS4A1*), neutrophils (*FCGR3B*) and erythrocytes (*HBB*). Neutrophils and erythrocytes totalized less than 50 cells in our whole dataset and were excluded from the analysis. We used Seurat AddModuleScore to search for other known cell-types based on gene-sets from a previous study of human lung atlas[75]. No additional cell-types were found in our BAL samples. We filtered low-quality cells and doublets based on the gene count per cell, where cells falling outside the interval of -1.5SD to + 2SD were excluded[76]. As the myeloid cells presented higher overall gene count per cell (~ 3k) compared to the lymphoid cells (~ 1k), the −1.5SD-+2SD gene count/cell filtering was done separately by main cell-type. Cells with more than 20% mitochondrial genes were also excluded as they were likely dead cells. Contaminated cells were excluded with DecontX[77] (implemented in Celda v1.10.0)[78]. Doublets were removed using DoubletFinder v2.0.3 using default parameters and manually curated based on co-expression of the canonical markers for the main cell-types[79]. At this step, four libraries prepared from one resister participant were excluded due to the high proportion of dead cells (**Supplementary Table 1**). Next, we combined the remaining 53 libraries that passed the pre-processing filtering (**Supplementary Table 1**). To help the integration and clustering by increasing the sample size, we included 10 in-house scRNA-seq libraries prepared from fresh BAL (these samples were not included in the downstream analyses and otherwise are not part of the results shown in this study). To integrate all libraries, normalization was done with SCTransform and integration with the RPCA method from Seurat v4.3.0[74,80]. This step was done with the top 1000 variable genes excluding mitochondrial and ribosomal genes[76]. For visualization, UMAP was used as dimensional reduction method, using the top 25 PCs.

## Cluster identification from the BAL cells

In the UMAP from all the BAL cells, we observed that the cells were separated into two main subsets of cells that were identified as lymphoid (T/NK/B cells) and myeloid (AM/DC) cells. To identify subpopulations of cells, the cells from the two main populations were separated based on the UMAP coordinates and each subset was re-integrated using the same method as the initial integration. Clustering was done with Seurat FindNeighbors and FindClusters functions. Parameters for the clustering were selected based on the cleanest separation found between T ($CD3D^{+}$) and NK ($CD3D^{-}$) cells in the lymphocyte subset and between TR-AM ($MARCO^{high}$) and MoAM/DC ($MARCO^{low}$) in the AM/DC subset. Hence, clustering was done using the first 25 PCs and resolution of 1.2 for the lymphocyte subset and of 0.8 for the AM/DC subset. An additional step of data cleaning was done to remove remaining low-quality cells and likely doublets of cells from the same main cell-type. For that, cells that were outliers in the UMAP for each cluster were removed. Three rounds of re-integration and cleaning were performed per subset. Libraries with less than 100 lymphocytes could not be included in the re-integration due to the low number of cells (**Supplementary Table 1**). We used Seurat FindTransferAnchors and TransferData functions with the lymphocyte re-integrated data as reference to annotate the lymphocyte clusters of the excluded samples, which was used for the cluster proportion estimates.

In total, we obtained 257,671 high-quality cells. The cluster annotation was based on three analyses done in parallel: i) we compared the expression of known canonical markers among the different clusters, ii) we compared the expression of the cell-surface markers from the two CITE-seq samples and iii) we performed a DE analysis to compare the gene expressions among clusters. To identify the DEG among clusters in the single cell data, we used Wilcoxon test as implemented by Seurat FindConservedMarkers function. The DE analysis was done between the cells from a cluster against all the remaining cells from the main population. This was done combining the cells from the two groups and incubation time-points but separated by the infection status. Genes were considered differentially expressed if presenting FDR < 0.05, expression in > 25% of the cells and absolute log2FC > 0.25 between the cluster and remaining cells in both tests: in the *Mtb*-infected and in the non-infected cells.

Comparison of the cell populations proportions and the CD4/CD8 T cell ratio between the LTBI and resister from BAL and PBMC samples were done using two-sided Wilcoxon tests with Bonferroni multiple test correction. We used box plots to present the population proportions by group, where the band in the box plot indicates the median, the box indicates the first and third quartiles and the whiskers indicate ± 1.5× interquartile range.

## Pseudobulk differential expression analysis

To perform differential expression analyses in the absence of *Mtb* (baseline), we created pseudobulk expression matrices and used linear models as implemented in packages for bulk RNA-seq. For that, the expression matrices were created separately for each cluster, where the gene expression counts per cell were aggregated by scRNA-seq library using Seurat AggregateExpression function. Libraries with less than ten cells in the cluster were excluded. Most libraries from the LTBI group did not pass this threshold for the lymphocyte clusters due to their low number of cells, which impeded the use of pseudobulk DE analysis in these cells. In the AM/DC subset, we performed the analysis for all the clusters except for the two smallest ones (AM.10 and AM.11). For the DE analysis in each cluster, genes were filtered in two steps: i) we excluded genes that were expressed in less than 10% of the cells from the cluster in both groups (SC-level expression matrices), and ii) we excluded genes that had pseudobulk count < 10 in more than 70% of the libraries (pseudobulk-level expression matrices). Libraries were normalized, scaled, and log2 transformed using edgeR v3.40.2 and Limma v3.54.2 (voom)[81–83].

We performed a differential expression analysis of the myeloid clusters between resisters and LTBI samples in the absence of *Mtb* (“baseline resister vs LTBI” analysis). For this analysis, we used all libraries per subject and removed the effects of infection and time-of-incubation by adding *Mtb*-infection status and hours of incubation as covariates in the model. In addition, we adjusted the analysis on the following variables to reduce confounding effects: length of HIV/ART of the patient, sequencing batch, fraction of dead cells during library preparation, number of cells aggregated for the pseudobulk expression. For quality control, principal component analysis (PCA) was calculated per cluster with prcomp function from stats v4.2.2 R package using the top 500 variable genes. Libraries from one BAL collected from a resister participant appeared as an outlier in the principal component analyses from the AM/DC clusters. These libraries were not outliers in the cell-types detection and proportions. Review of the pipeline from sample preparation suggested possible BAL cell contamination. Hence, these libraries were excluded from the downstream analyses to avoid artifacts in the DE tests caused by cross-contamination (**Supplementary Table 1**). The results of the “baseline resister vs LTBI” analysis were presented as the log2FC of the gene expression between resister and LTBI cells in the absence of *Mtb*. For multiple test correction, we used the Benjamini-Hochberg false discovery rates (FDR). Genes were considered differentially expressed when presenting absolute log2FC > 0.2 and FDR < 0.1.

## GO-term/pathway enrichment analysis

To identify GO-terms and pathways enriched in the DEG for each analysis, we used enrichGO function for GO-terms and enrichKEGG for KEGG pathways as implemented by clusterProfiler v4.6.2 R package[84]. We also searched for enriched Reactome pathways using enrichPathway function from ReactomePA v1.42.0 package[85]. For multiple test correction, we calculated the Benjamini-Hochberg FDR on the list with GO-term, Reactome and KEGG combined. Enrichment analysis was done for DEG with negative and positive log2FC separately and combined. GO-terms and pathways were considered significant if presenting FDR < 0.05 and count of ≥ 5 DEG. To represent a gene-set overall expression by sample, we calculated the module score by adapting AddModuleScore function from Seurat to use in the pseudobulk expression matrix. With this function, the average expression of a selected gene-set was calculated and subtracted by the aggregate expression of random control feature sets[86].

## Transcription factor activity score

Transcription factor activities were inferred per cell given the list of DEG per myeloid cluster detected in the pseudobulk DE analyses. TF analysis was done using the non-infected cells with 6h of incubation. For that, a Univariate Linear Model (ULM) was used to test the TF activity per cell using decoupleR v2.8[87]. For each cell, we calculated a TF t-score based on the linear correlation of gene expression and TF-gene interaction weights. For the TF-gene interaction we used CollecTRI[88], a curated collection of TFs and their corresponding targets, and tested activity for TF that had at least five DEG as their targets. To assess TF-activity per myeloid cluster, we calculated the mean t-score and standard deviation from the cells in the cluster. The mean t-scores were calculated separately in the cells from the resisters and LTBI in the absence of *Mtb*. A t-test was then used to evaluate significant differences in mean TF-activity per cluster between cells from the two groups. A Benjamini-Hochberg correction was applied to calculate the FDR for all tested TF and AM/DC clusters. TF displaying FDR < 0.01 and absolute difference of normalized TF-score > 0.2 were considered significant. For visualization, heatmaps were created using ComplexHeatmap v2.14 package.

## Cell-cell communication

We used CellChat v1.6.1 package to investigate the cell-cell communication network between the BAL cell population from the resister and LTBI samples[89]. Cellular communication was inferred based on the expression of known ligand-receptor pairs in the non-infected BAL cells with 6h of incubation, separately for the resister- and LTBI-derived cells. For that, we used the list of known human ligand-receptor network from CellChatDB database[89]. To compute the communication probability, mean average expressions were calculated with the default “triMean” method from CellChat. Clusters with less than 10 cells were excluded. We compared the number of interactions per signaling pathway between the two groups using

Wilcoxon test with a threshold of $P < 0.05$ and plotted the results using CellChat rankNet function. Finally, CellChat functions for circle plots were used to present the network of specific signaling pathways or specific ligand-receptor pairs in the groups.

#### Differential expression analysis of response to ex vivo Mtb challenge

We analyzed the transcriptomic expression changes and positive cell proportions changes of selected anti-*Mtb* genes in the infected and non-infected myeloid and lymphoid cells from the two post-infection time-points: 6h and 24h. Cells from libraries that fell out of the 5-7h and 22-25h incubation ranges were excluded (**Supplementary Table 1**). Gene expressions of selected genes were compared from the single-cell expression matrices using Seurat FindMarkers function with default settings. Eight pairwise comparisons were performed per cluster aiming to detect if a gene changed expression with infection in a group and if expression after infection was different between the groups. For that, we compared i) non-infected cells from the resister vs LTBI cells by time-point (two contrasts), ii) *Mtb*-infected cells from the resister vs LTBI cells by time-point (two contrasts), and ii) *Mtb*-infected vs non-infected cells by group by time-point (four contrasts). Clusters with less than 10 cells in a specific group and condition were excluded. DE was done only if the gene was expressed in > 10% of the cells in at least one of the contrasted group of cells. Absolute log2FC > 0.1 and Wilcoxon $P < 0.05$ were used as thresholds. For visualization, we used Seurat functions VlnPlot and DotPlot.

## Declarations

#### Data availability

The raw and processed BAL scRNA-seq data from the 6 resisters and 7 LTBI participants are being deposited to the Gene Expression Omnibus (GEO) and will be available at the time of publication.

#### Funding

This work was supported by a grant from the National Institutes of Health (NIH) to E.S., N.dP., E.H. (Grant R01AI124349) and to S.B-D., L.A., and J-L.C. (U19AI162568 and UL1TR001866). In addition, this work was funded by a grant from the Canadian Institutes of Health Research (CIHR) to E.S. (Grant FDN-143332). This research was supported through resource allocation in Cedar high performance computing clusters by Digital Research Alliance of Canada. N.dP. and G.W received funding from (NIAID), NIH, through contract #75N93019C0070 and 12446SUB. The Laboratory of Human Genetics of Infectious Diseases of L.A. and J-L.C. was supported by the French National Research Agency (ANR) under the “Investments for the Future” program (ANR-10-IAHU-01), the St. Giles Foundation, and the General Atlantic Foundation. S.B-D. received funding from the French National Agency for Research on AIDS and Viral Hepatitis (ANRS) (ECTZ170784-ANRS0073). We acknowledge the support of the South African Medical Research Council Centre for Tuberculosis Research, Division of Molecular Biology and Human Genetics, Faculty of Medicine and Health Sciences, Stellenbosch University, Cape Town, South Africa. The funders played no role in the study design, data collection and analysis, decision to publish or preparation of the manuscript.

#### Acknowledgments

We sincerely thank the study participants who accepted to be enrolled in the study and to undergo broncho alveolar lavage. We also thank all team members of the ResisTB study. Special thanks to Stellenbosch University clinical team for valuable support in sample collection and transportation and the laboratory team members Stephan Botha, Devon Allies, Ayanda Shabangu for sample processing, storge and management. We thank the personnel of the CL3 Facility at the RI-MUHC (Montreal). As a multi-centric study, we thank all members of the different laboratories involved in the study – Schurr lab at McGill University (Montreal), Möller, Hoal, du Plessis and Walzl labs at Stellenbosch University (Cape Town), Abel lab at the Imagine Institute (Paris) and Casanova lab at the Rockefeller University (New York) – for useful comments and suggestions to the study and writing of the manuscript. We thank Wilian Correa-Macedo, Dr. David Langlais, Dr. Mathieu Mancini, Dr. Mathieu Bourgey, Dr. Pauline Rivière, Masato Ogishi, Dr. Tracey Jooste and Maynard Meiring for their useful comments and info for the data analysis. We thank Dr. Yong Zhong Xu and Pauline Cassart for their technical support in the wet-lab. Finally, we thank Dr. Marcel Behr from McGill University for his comments on earlier versions of the manuscript.

#### Contributions

M.D-S., V.M.F., M.O., A.C., E.G.H., L.A., M.M., J-L.C., G.W., N.dP. and E.S. performed study design and conceptualization. M.D-S. performed the scRNA-seq data processing and analysis. V.M.F. performed data analysis for TF and cell-cell communication. S.T.M and C.McD. performed clinical procedures. S.T.M, C.McD., E.E.K. and M.M. recruited and enrolled subjects. M.O. performed the cell-based experiments for the scRNA-seq. M.M. and E.S. performed project administration. G.W. and N.dP. performed data collection. E.G.H., N.dP. and E.S. produced funding sources. E.S. supervised the project. M.D-S., V.M.F., M.O., A.C., S.B-D., E.G.H., L.A., M.M., J-L.C., G.W., N.dP. and E.S. contributed to the data interpretation. M.D-S. and E.S. wrote the draft of the manuscript and V.M.F., S.T.M., M.O., A.C., S.B-D., L.A., M.M., J-L.C., G.W. and N.dP reviewed and edited it. M.D-S. and V.M.F. worked on visualization. All authors read the final manuscript.

## Figures

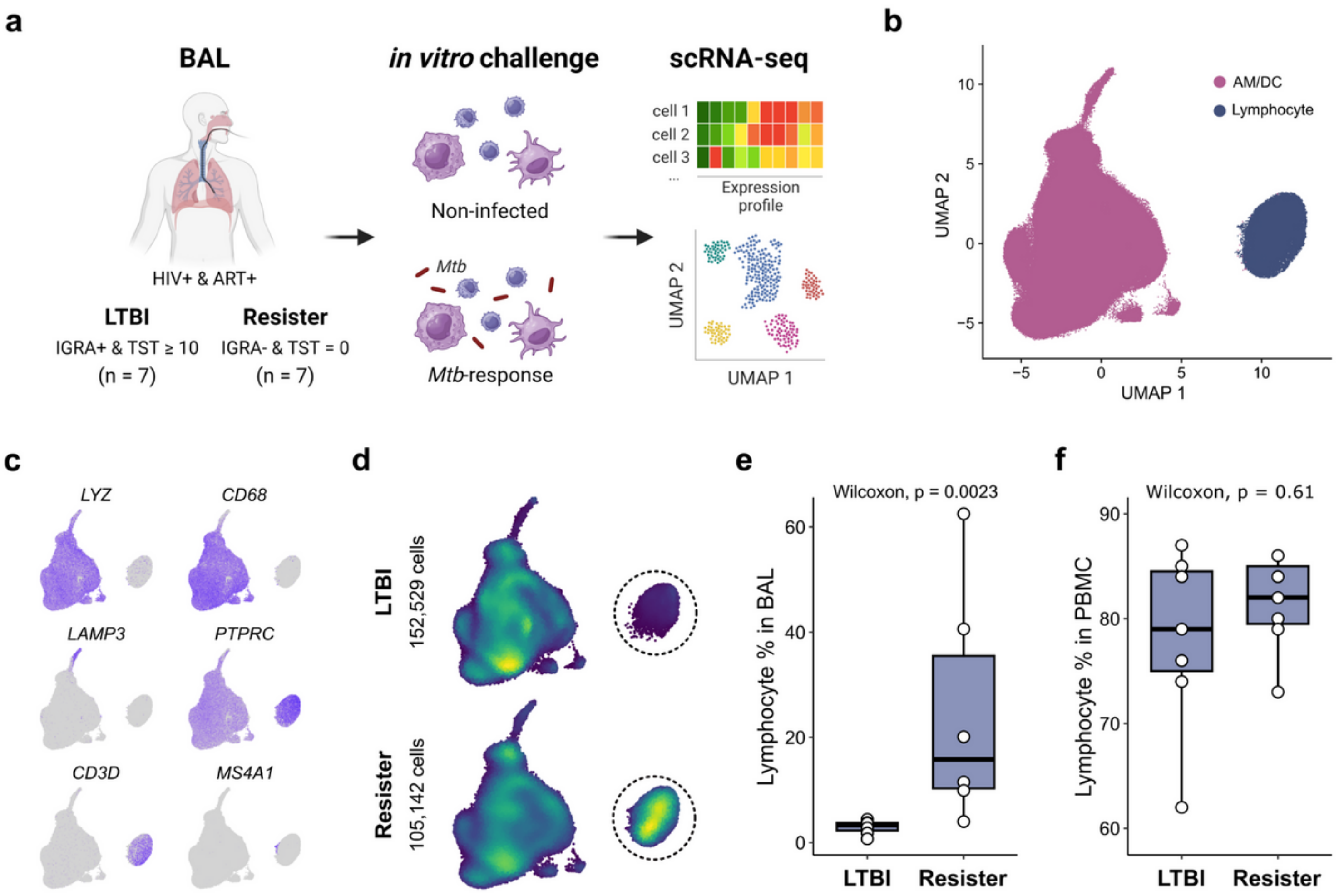

### Figure 1

#### Resisters have higher lymphocyte proportion in cells obtained by BAL compared to LTBI.

**a,**Schematic representation of the study design. BAL cells were obtained from all study participants and scRNA-seq was conducted at 6h and 24h in the presence and absence of *Mtb* infection. Gene expression data were derived both for uninfected (operationally defined as baseline) and infected BAL cells. Analysis of scRNA-seq data was used to estimate BAL cell identities and proportions and to perform differential expression analysis. **b,** Uniform Manifold Approximation and Projection (UMAP) of the scRNA-seq data from the BAL cells of all subjects identified alveolar macrophages (AM)/Dendritic cells (DC) and lymphocytes as main populations. **c,** Gene expression of canonical markers for macrophages (*LYZ* and *CD68*), DC (*LAMP3*), leukocytes (*PTPRC* [CD45]), T cells (*CD3D*) and B cells (*MS4A1*). Higher expressions are shown by darker colors in the UMAP. **d,** Density of cells obtained from LTBI and resister participants. Yellow and dark blue colors indicate the highest and lowest density of cells in the UMAP, respectively. UMAPs included samples irrespective of infection status and incubation time-point. **e,**Box plot of lymphocyte proportions (%) in BAL cells obtained from resister and LTBI participants. Each dot represents the average lymphocyte percentage obtained from the scRNA-seq libraries per subject. **f,** Box plot of lymphocyte proportion (%) in peripheral blood mononuclear cells (PBMC) for the same resister and LTBI participants.

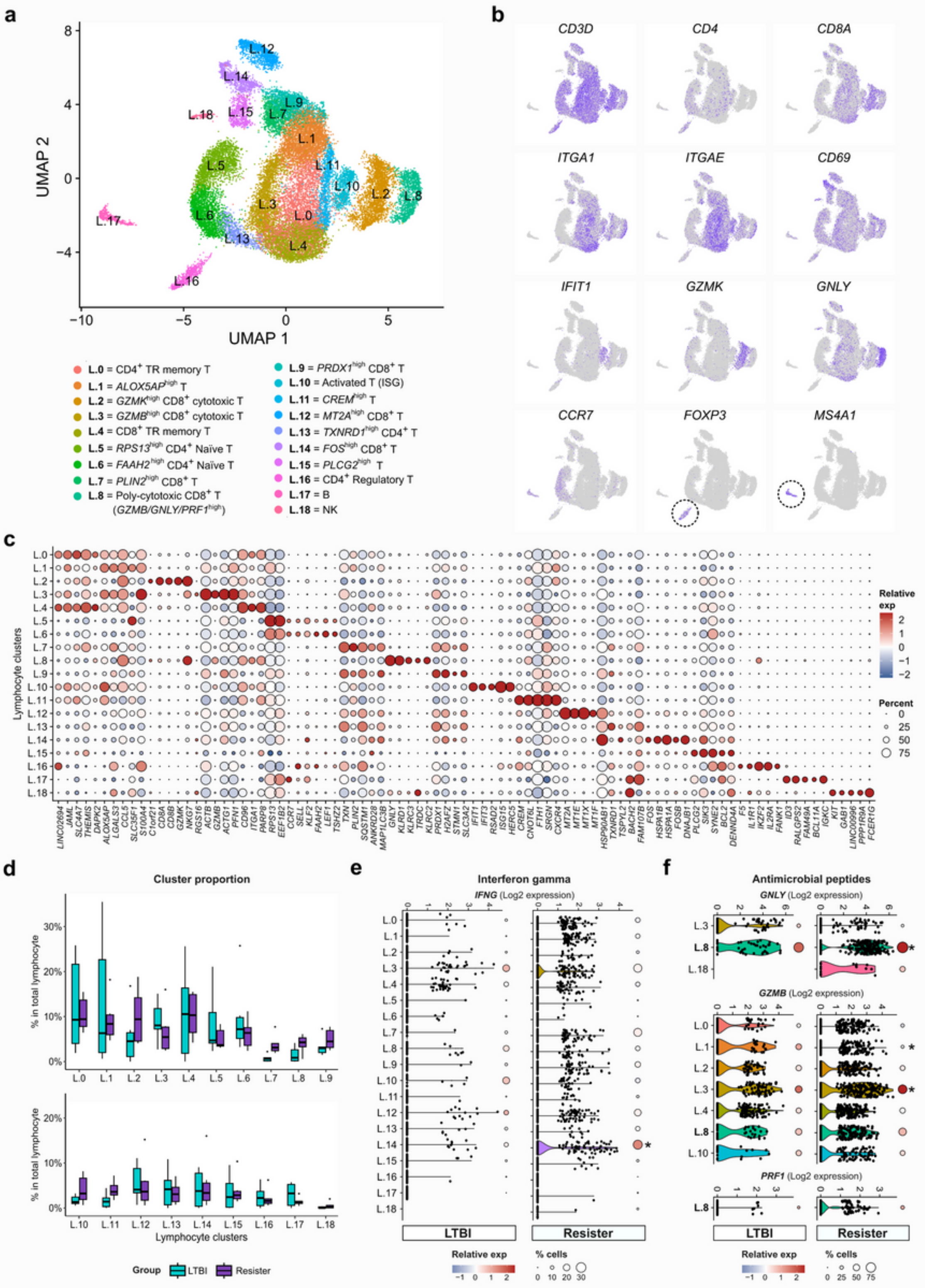

### Figure 2

#### Identification of lymphocyte subpopulations in BAL.

**a,**UMAP of the lymphocyte subset showing 19 lymphocyte clusters and their annotations. Data from both groups with samples from all conditions. TR: Tissue-resident. **b,** UMAP showing the gene expression of selected canonical markers used for the annotation of lymphocyte clusters. Higher expression is reflected by darker colours. **c,** Dot plot of the top five genes with higher expression for each cluster compared to the remaining lymphocytes. Gene names are identified at the bottom of the graph and the lymphocyte clusters are shown by rows. Colour and size correspond to the scaled expression and the percentage of cells expressing the gene by cluster, respectively. Data from non-infected samples. **d,** Box plot of lymphocyte cluster proportions relative to total alveolar lymphocytes from resister and LTBI samples. Data from 6h non-infected samples. Two clusters presented nominal $P < 0.05$ using a two-sided Wilcoxon test (L.7 $P = 0.008$ and L.11 $P = 0.045$) but failed to pass multiple test correction (Bonferroni threshold: $P <$ 0.0026 [ 0.05/19]). **e,** Violin plots of *IFNG* expression by 6h non-infected cells for resister and LTBI lymphocyte clusters. Each dot represents a single cell. The size of the circles on the right of each violin indicates the proportion of cells expressing *IFNG*. Circle colours indicate a scaled *IFNG* expression in positive cells relative to all lymphocytes in the cluster. The asterisk indicates a significant *IFNG* expression difference (Wilcoxon $P < 0.05$) between resister and LTBI clusters. Cluster L.18 in the LTBI group had less than 10 cells and is not plotted. **f,** Violin plots of the transcripts of antimicrobial peptide *GNLY*, *GZMB* and *PRF1*in non-infected lymphocytes from LTBI and resisters. Figure legend as detailed in panel “e”. For each gene, only the clusters presenting ≥25% of positive cells in at least one of the groups are shown.

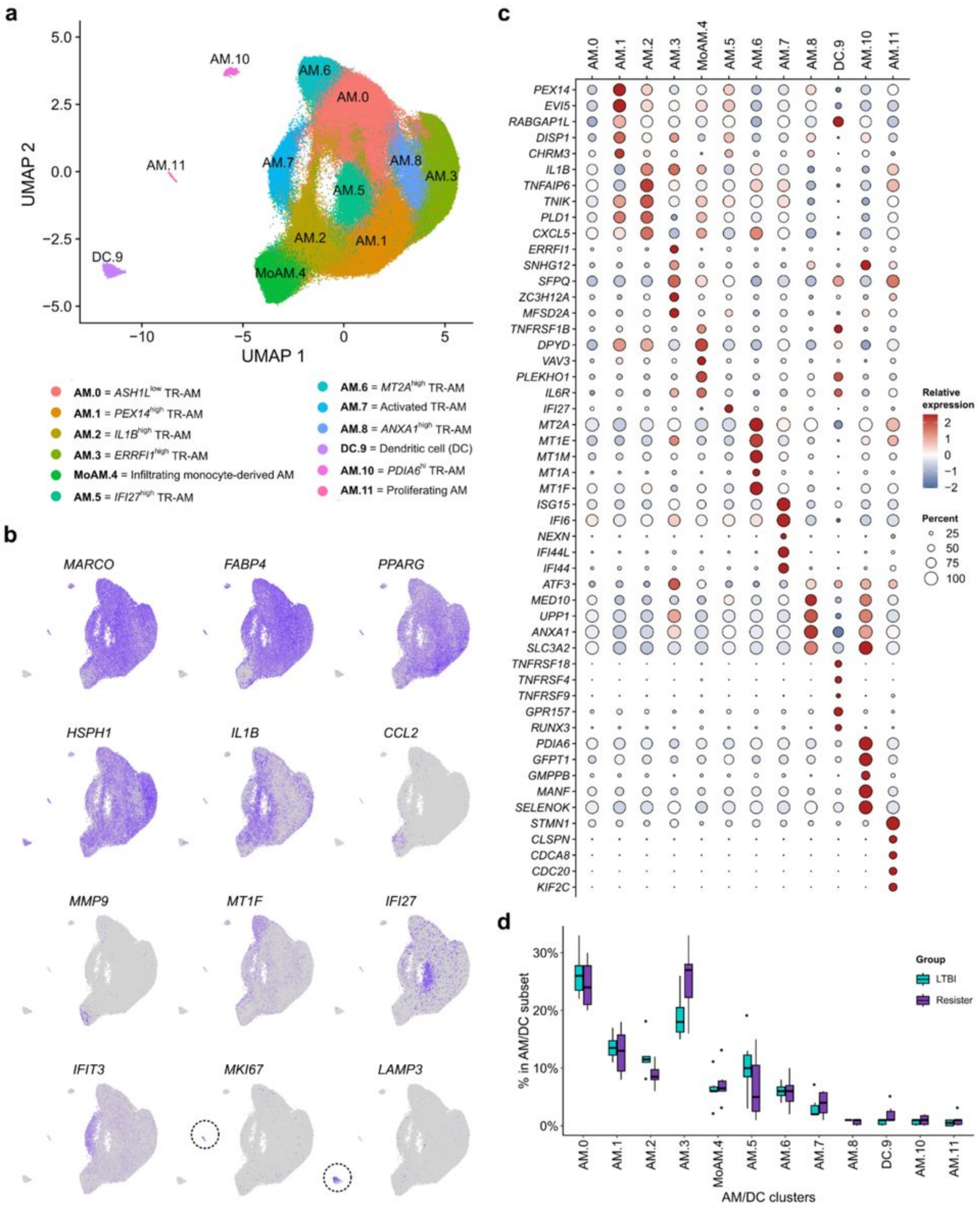

### Figure 3

#### Identification of AM/DC subpopulations in BAL.

**a,**UMAP of the AM/DC subset showing 12 clusters and their annotations. AM: Alveolar macrophages, DC: dendritic cells, TR: Tissue-resident. **b,** UMAP showing the gene expression of selected canonical markers used for the annotation of the AM/DC clusters. Gene names are shown on the top of the UMAPs. Higher expressions are shown by darker colours. For UMAPs, data from all samples and all conditions. **c,** Dot plot of the top five genes with higher expression compared to the remaining AM/DC cells for each cluster. Colour and size correspond to the scaled expression and the percentage of cells expressing the gene by cluster, respectively. Data from non-infected samples. **d,** Box plot displaying cluster proportions relative to the total myeloid population from resister and LTBI BAL samples. Data from 6h non-infected samples. Two clusters presented nominal $P < 0.05$ using a two-sided Wilcoxon test (AM.3 $P = 0.041$ and DC.9 $P = 0.026$) but failed to pass multiple test correction (Bonferroni threshold: $P < 0.0042$ [ 0.05/12]).

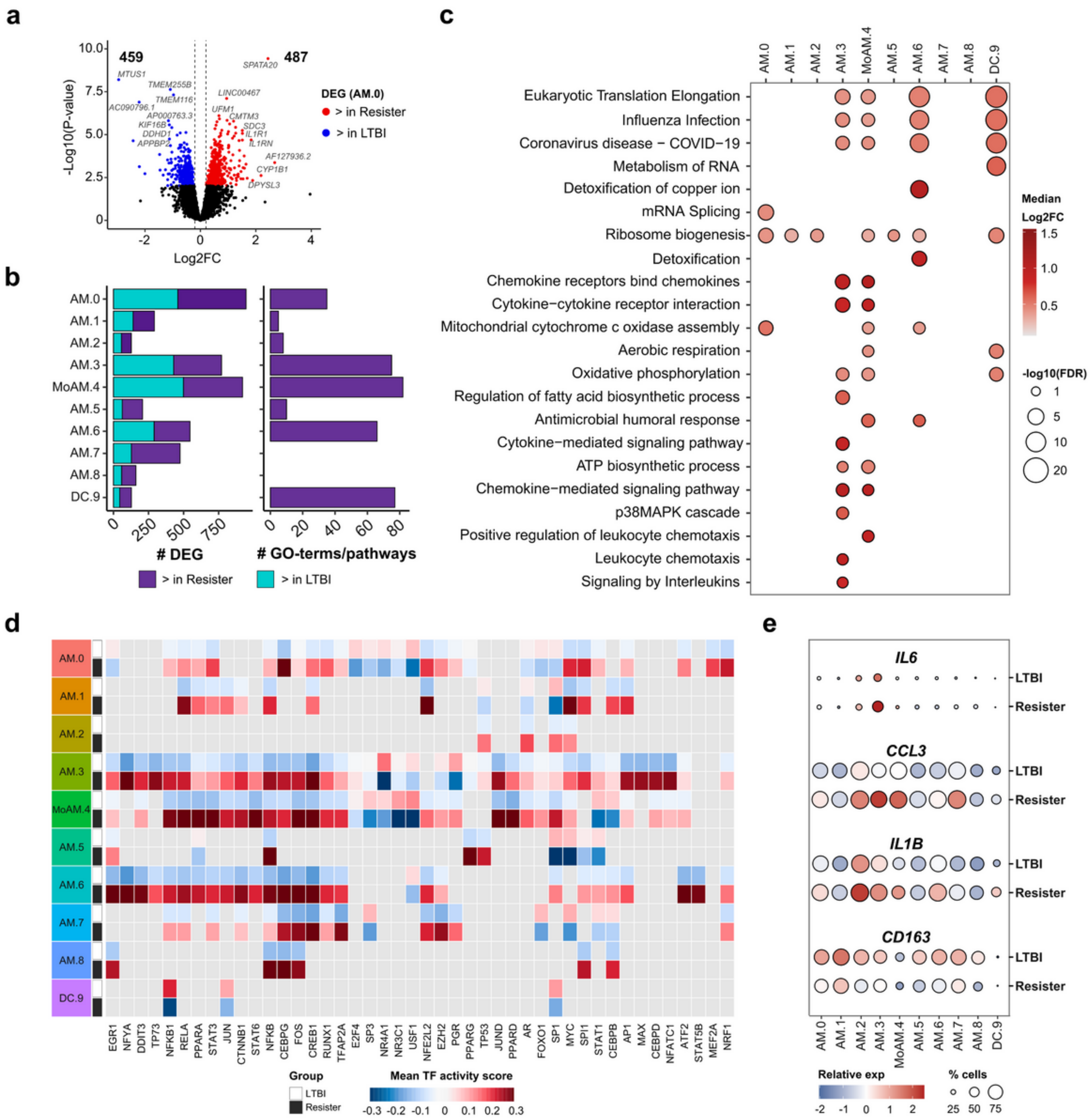

### Figure 4

**Gene expression differences in the absence of *Mtb* between resister and LTBI AM/DC subpopulations.**

**a,**Volcano plot for differences in gene expression between resister and LTBI samples for subpopulation AM.0, in the absence of *Mtb*. Volcano plots of remaining tested clusters are shown in **Extended Data Fig. 4**. The x-axis shows the log2 fold-change (log2FC) difference of gene expression between resister and LTBI samples. The y-axis presents the -log10 of the unadjusted *P*-value for significance of expression differences. Dashed lines correspond to the log2FC thresholds of -0.2 and 0.2. DEG with higher expression in cells from resisters or LTBI are shown in red and blue, respectively (FDR < 0.1). Total numbers of DEG higher or lower expressed in resister samples are indicated in the top corners. **b,** Left panel: bar plots of the numbers of DEG across all AM/DC subpopulations. Purple and light blue indicate DEG with higher or lower expression in cells from resister vs LTBI participants, respectively. Right panel: Numbers of GO-terms/pathways in which DEG are enriched. The only significant terms detected were for DEG with higher expression in resister samples. **c,** Dot plot of selected enriched GO-terms/pathways from genes with higher expression in AM/DC from resister compared to LTBI samples. The size of a circle corresponds to the significance of DEG enrichment in the GO-term/pathway. The colour represents the median log2FC expression difference of term DEG between resister and LTBI samples. Dots are only shown in AM/DC subpopulations with FDR < 0.05 for enrichment of DEG in the term. **d,** Heatmap of differential transcription factor (TF) activity in resister and LTBI BAL samples for 6h non-infected cells. The heatmap shows the top 10 TF displaying the largest mean differential activity per AM/DC cluster, except for clusters with less than 10 significant TF. The mean TF activity scores for cells in each cluster are shown for the LTBI (white box) and resister cells (black box). Positive scores indicate stronger TF-activity and negative scores indicate weaker/inactive TF-activity. Non-significant (FDR > 0.01) or not tested TFs are shown in grey, details in methods. **e,**Dot plot with gene expression of M1 genes *IL6*, *CCL3* and *IL1B*, and the M2 gene *CD163* in the non-infected cells with 6h of incubation. For each gene, the expression of LTBI and resister-derived cells are shown in the first and second row, respectively. The size of the circles indicates the percentage of cells expressing the corresponding gene. The shade of the circles shows the expression levels of the indicated gene relative to all other clusters. Darker red or blue colours indicate higher and lower scaled expressions, respectively.

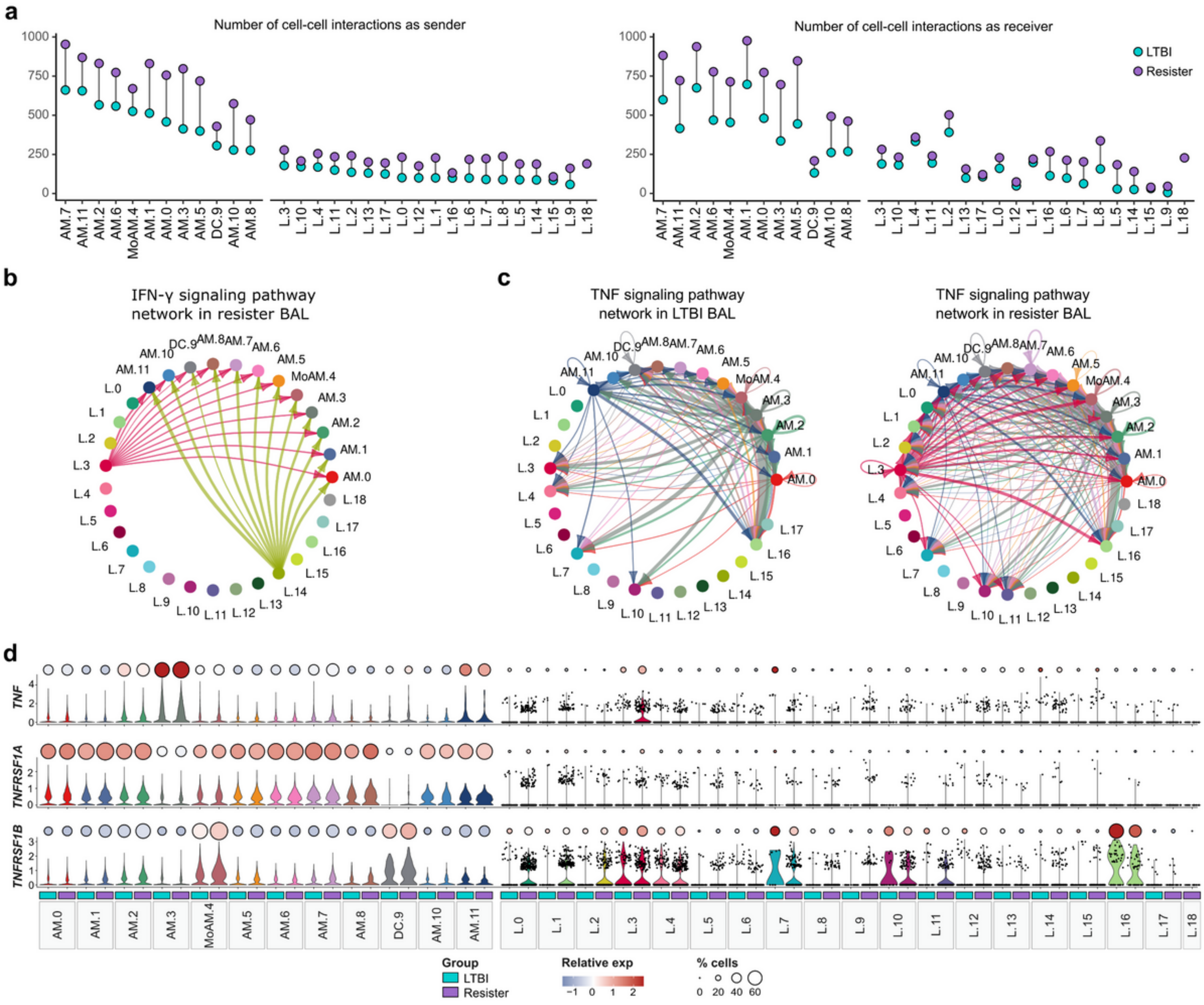

### Figure 5

#### Cell-cell communication differences between BAL cells from resister and LTBI samples in the absence of Mtb.

**a,**Total number of cell-cell interactions per cluster. The left plot displays the number of cells expressing the ligand genes (senders). The right plot displays the number of cells expressing the receptor genes (receivers). Number of interactions for resister and LTBI cells are shown in purple and light blue, respectively. Clusters are ordered based on the number of interactions as senders in the LTBI group. **b,**Circle network diagram of cell-cell interactions for IFN-g signaling which was detected only in resister samples. The nodes represent the cell clusters, and the edges represent the interactions. The arrows indicate the sender → to receiver interactions, which for IFN-gwere exclusively from T cells to AM/DC. The thickness of the edge denotes the interaction strength. **c,** Circle network diagram of cell-cell interactions for TNF signaling found in the LTBI (left) and resister samples (right) including both TNF -TNFR1

(*TNFRSF1A*) and TNF - TNFR2 (*TNFRSF1B*) receptors. Results of individual TNFR1 and TNFR2 cell chats are presented in **Extended Data Fig. 6c-d**. Nodes and edges legends as in panel "b". **d,** Violin and dot plot of *TNF* gene expression as the ligand and the genes *TNFRSF1A* and *TNFRSF1B* encoding its receptors. Each cluster is divided by resister and LTBI samples (teal and lilac boxes, respectively). The size of the circles indicates the percentage of cells in each cluster that express the corresponding gene. The shade of the circles indicates the expression levels of each gene relative to all other clusters. Due to the high number of myeloid cells, individual points (cells) are not shown. **a-d,** Data from 6h non-infected samples. Cluster L.18 in LTBI group was excluded due to having < 10 cells in total.

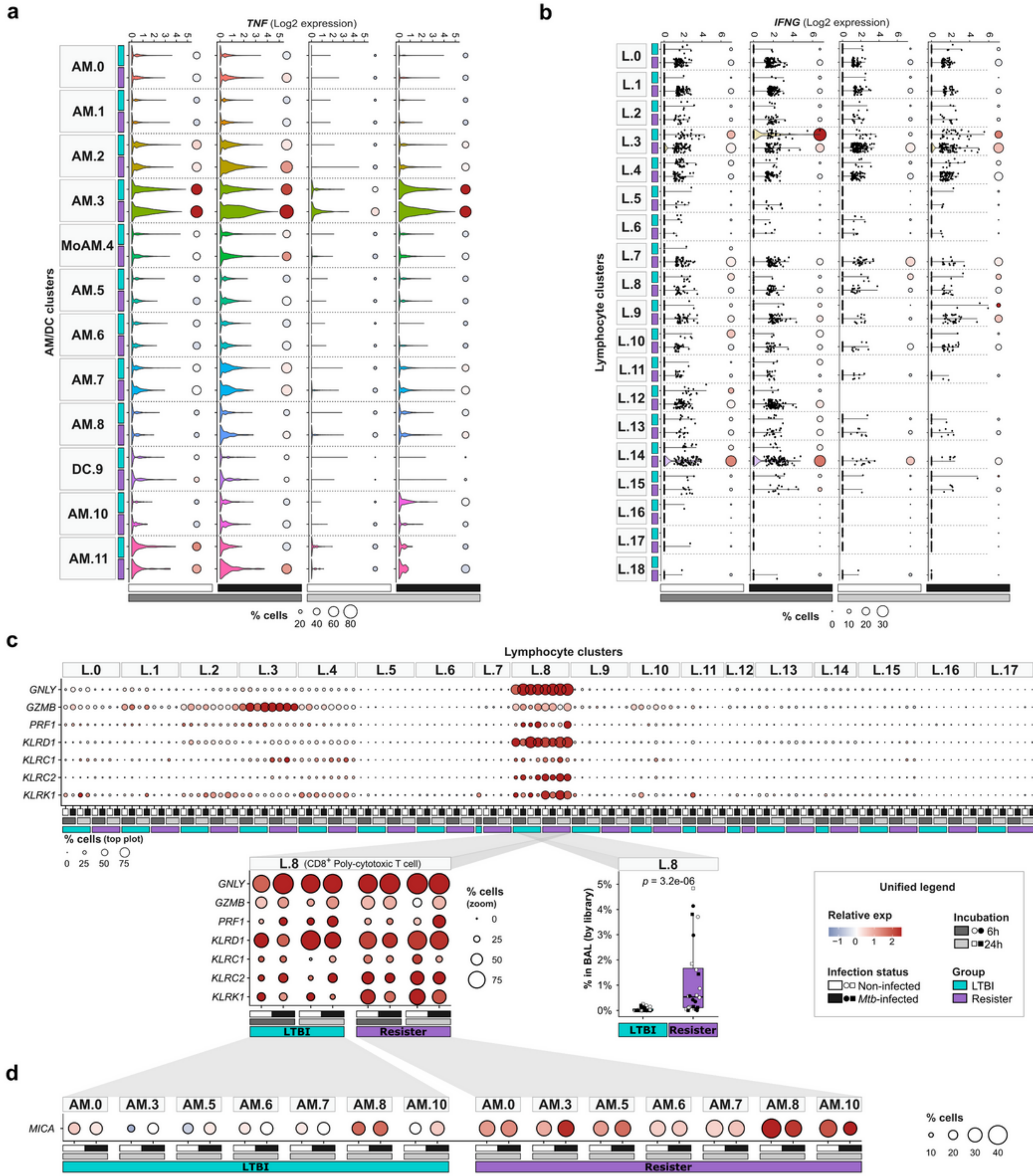

### Figure 6

### Alveolar immune cell responses to infection with Mtb.

**a,** Kinetics of *TNF* transcription at 6h and 24h of *in-vitro*culture in presence and absence of *Mtb* in LTBI and resister myeloid clusters. The violin plots present the density and distribution of the *TNF*log2 expression (x-axes) in the different clusters by group (y-axes) and condition (by column). Dot plots are presented on the right of the violins. For each cluster, the size of the circles indicates the percentage of cells expressing *TNF*. Circle colours indicate *TNF* scaled expression of the *TNF*-positive cells from the different clusters and conditions. Size legend is shown on the bottom and the colour legends are presented in panel "c". TNF transcription by lymphoid cells did not change significantly and is not included in the graph. The violin colors represent the different myeloid clusters. **b,** Kinetics of *IFNG* transcription at 6h and 24h of *in-vitro* culture in presence and absence of *Mtb*in LTBI and resister lymphocyte clusters. Violin and dot plot legends are as detailed for panel "a". Each dot in the violin plots represents a cell. For each group and condition, clusters with less than 10 cells were excluded. The violin colors represent the different lymphoid clusters. **c,** Kinetics of antimicrobial peptides *GNLY*, *GZMB*, *PRF1* and NK receptors *KLRD1*, *KLRC1, KLRC2* and *KLRK1* transcription at 6h and 24h of *in-vitro*culture in presence and absence of *Mtb* in LTBI and resister lymphocyte clusters. Violin and dot plot legends are as detailed for panel "a". The dot plot break-out insert below the lymphocyte cluster graph focuses on the changes of the seven genes in cluster L.8. To the right of the break-out is a box plot of estimates of the L.8 cell frequencies in BAL samples from all resister and LTBI libraries (see Methods). Significance of the difference in proportion between the groups was calculated using Wilcoxon test and the *P*-value is shown on top of the box plot. **d,** Kinetic of the *MICA*transcription at 24h of *in-vitro* culture in presence and absence of *Mtb*in selected AM clusters from resister and LTBI participants.

## Supplementary Files

This is a list of supplementary files associated with this preprint. Click to download.

- CTSCSupplTable1.xlsx
- CTSCSupplTable2.xlsx
- CTSCSupplTable3.xlsx
- CTSCSupplTable4.xlsx
- CTSCSupplTable5.xlsx
- CTSCSupplTable6.xlsx
- CTSCSupplTable7.xlsx
- SchurrFlatEPC.pdf
- SchurrFlatRS.pdf
- Extendeddata.docx
